# Magnitude and reasons for pre-diagnosis attrition among presumptive multi-drug resistant tuberculosis patients in Bago Region, Myanmar: A mixed methods study

**DOI:** 10.1038/s41598-019-43562-3

**Published:** 2019-05-10

**Authors:** Tun Oo, Khine Wut Yee Kyaw, Kyaw Thu Soe, Saw Saw, Srinath Satyanarayana, Si Thu Aung

**Affiliations:** 1 Assistant Director, National Tuberculosis Programme, Bago Region, Ministry of Health and Sports, Bago, Myanmar; 2 Operational Research Fellow, Department of Operational Research, International Union Against Tuberculosis and Lung Disease (The Union), Mandalay, Myanmar; 3 Research Officer, Department of Medical Research (Pyin Oo Lwin branch), Ministry of Health and Sports, Pyin Oo Lwin, Myanmar; 4 Director (Planning), Department of Medical Research, Ministry of Health and Sports, Yangon, Myanmar; 5 Deputy Director, Center of Operational Research, International Union Against Tuberculosis and Lung Disease (The Union), Paris, France; 6 Program Manager, National Tuberculosis Programme, Ministry of Health and Sports, Nay Pyi Taw, Myanmar

**Keywords:** Epidemiology, Epidemiology, Epidemiology, Outcomes research, Outcomes research

## Abstract

In Myanmar, Rifampicin resistant tuberculosis (RR-TB, a proxy for Multi-drug resistant TB) case detection is very low. Our study objectives were to assess the proportion of eligible TB patients who had not undergone RR-TB testing (Xpert-MTB/Rif tests) in Bago Region, Myanmar and to understand the reasons and solutions for non-testing. We conducted a mixed-methods study involving analysis of routinely collected programme data followed by key informant interviews (KIIs) with 32 health care providers. From October 2016 to March 2017, of the 2,331 eligible patients, 1,066 (46%) had not undergone Xpert-MTB/Rif testing. Patients from townships without Xpert-MTB/Rif testing facilities, new TB patients, patients whose HIV status was negative or unknown and extra pulmonary TB patients were less likely to undergo Xpert-MTB/Rif testing. From the health care providers’ perspective, the most common reasons for non-testing were: (a) lack of awareness of the eligibility criteria; (b) difficulties in collecting sputum and transportation from eligible patients to the testing sites. We conclude that nearly half of eligible patients were not tested for RR-TB. Training of health care providers about the latest eligibility criteria and improvement in sputum collection and transportation systems particularly for townships without Xpert-MTB/Rif testing facilities are required to improve RR-TB testing.

## Introduction

Multi-drug resistant tuberculosis (MDR-TB), i.e., infection with *Mycobacterium tuberculosis* that is resistant to the two most potent anti-TB drugs namely, rifampicin and isoniazid, is an important challenge for TB control worldwide. Early detection and prompt treatment of patients with MDR-TB is crucial to reduce morbidity, mortality and transmission in the community. However, globally in 2017 only 29% of an estimated 558,000 incident MDR-TB patients were detected and of those detected, 87% were initiated on treatment^1^. This indicates that the national tuberculosis programmes in high TB burden countries encounter challenges in the diagnosis and treatment initiation of MDR-TB^2^.

Operational challenges in diagnosing and initiating patients on MDR-TB treatment are reflected in two attritions, namely ‘pre-diagnostic’ and ‘pre-treatment attrition’^3^. “Pre-diagnostic attrition” indicates failure to identify and test eligible patients with presumptive MDR-TB with MDR-TB laboratory tests, and “pre-treatment attrition” reflects failure to initiate diagnosed MDR-TB patients on MDR-TB treatment. It is recommended that the TB programmes in all high burden countries monitor such attritions, identify the reasons and undertake corrective measures to improve care provided to these patients^4,5^.

Myanmar is one of the 30 high MDR-TB burden countries with an estimated annual incidence of 13,000 MDR-TB cases (incidence rate = 25/100,000 population) in 2016^2^. The prevalence of MDR-TB among new and retreatment TB cases is about 5% and 27% respectively, which is higher than global average of 4.1% and 19% in 2016^2^. Services for MDR-TB diagnosis and treatment were initiated under the National TB Programme (NTP) in 2009 as a “DOT-Plus” pilot project in 10 townships, which was later renamed as “Programmatic Management of Drug-resistant TB” (PMDT) in 2011^6^. During the initial years, the diagnosis of MDR-TB was based only on results of sputum culture and drug sensitivity testing. This could be done only in two laboratories (located at Yangon and Mandalay), and therefore due to limited access and long turnaround time to obtain results, anecdotal evidence indicates that the number of MDR-TB patients diagnosed and initiated on treatment was low. With the introduction and scale up of rapid diagnostic tests [Xpert MTB/Rif ® tests, called ‘GXP tests’ henceforth] and with this test providing results on the presence of *Mycobacterium Tuberculosis Complex* and rifampicin resistance (RR-TB, rifampicin resistant TB) within 2 hours with relatively high sensitivity and specificity^7^, it was expected that the situation would improve. By the end of 2017, 71 GXP machines were available at district TB centers in various parts of the country. For all practical purposes, in order to simplify the case management under routine programmatic conditions, RR-TB patients are considered as proxy for having MDR-TB in Myanmar.

Though GXP testing sites have increased in numbers, diagnosing all RR-TB patients are still a major challenge for the NTP. As per the National Strategic Plan (NSP) 2016–2020, the Myanmar NTP had set a target of notifying 4,816 RR-TB cases in 2016^8^. However, in 2016 only 3,213 RR-TB cases were notified —a short fall of 1,603 (33%) cases (2016 NTP data). The reasons for the shortfall were not completely known and anecdotal evidence indicated that the reasons vary across different states and regions of the country. A previous study from Yangon and Mandalay regions showed high pre-treatment attrition rates (30%)^9^. But there have not been any studies describing the magnitude and reasons for pre-diagnosis attrition in Myanmar (definitions of pre-treatment and pre-diagnosis attrition are mentioned in the second paragraph). Recently, in order to accelerate the RR-TB case finding in the country, the NTP has set targets for testing 70% and 75% of all eligible presumptive RR-TB cases in 2016 and 2017 respectively by GXP tests.

Bago Region in Myanmar has a population of 5 million (~10% of Myanmar’s population)^10^. If the national RR-TB prevalence estimates are applied to the TB patients diagnosed in Bago region, in 2016, about 550 RR-TB cases (177 RR-TB patients among 3,552 new smear positive TB cases and 372 RR TB patients among 1,373 retreated TB cases) should have been detected. However, only 179 (33%) of the estimated 550 RR-TB patients were detected in 2016 indicating that either the RR-TB prevalence is low in the region or that the TB programme is not able to diagnose RR-TB cases. [2016 Bago Region TB data, personal communication]

We hypothesized that low RR-TB detection in Bago region is more likely due to pre-diagnosis attrition. This is because, the population in Bago Region is sparsely distributed, is predominantly rural in nature and the numbers of GXP testing machines/facilities are few. Therefore, we undertook an operational research study to understand the magnitude and reasons for pre-diagnosis attrition.

The specific objectives were as follows. Among TB patients enrolled for first-line anti-TB treatment under the NTP in Bago Region between October 2016–March 2017: first, to assess the number (and proportion) who were presumptive RR-TB patients and eligible for GXP testing, and of those eligible, to determine the number (and proportion) who underwent GXP testing and their demographic, clinical characteristics; second, in those who had undergone GXP testing, to assess the duration between date of eligibility for GXP testing and date of GXP testing; and third, to understand the health provider perspectives on the reasons for failure to identify and test eligible TB patients with GXP tests.

## Methods

### Study design

This was a sequential explanatory mixed methods study beginning with the quantitative phase and then the qualitative phase, to explain or enhance the quantitative results^11^. For the first two objectives (quantitative phase) we used a retrospective cohort study design and involved secondary analysis of data routinely collected by NTP. For the third objective, we used a descriptive study design (qualitative phase) and conducted key informant interviews (KIIs) with 28 township TB coordinators and laboratory technicians of 4 GXP sites in Bago Region.

### Setting

Myanmar is a low middle income South-East Asian country with population of ~51 million. There are 74 districts (330 townships)^10^. About 70% of the population lives in rural areas. Health coverage of rural population is predominantly provided through primary health care facilities.

The Myanmar NTP is managed by a team lead by a Program Manager at the central level in Nay Pyi Taw and TB Officers at regional and district levels. TB diagnostic and treatment services are integrated into the public health care system. Each township in the country has a township TB coordinator who is primarily responsible for coordinating all the activities of NTP (diagnosis, treatment, recording and reporting, drug management etc.). TB cases are predominantly diagnosed by sputum smear microscopy and chest radiography. All detected TB cases are classified using standard WHO recommended case definitions and enrolled in the “paper based TB registers” of the respective township TB center for TB treatment as per NTP guidelines^12^. Prior to initiating the patients on TB treatment, certain groups of TB patients are classified as “presumptive RR-TB patients” and these patients are eligible to undergo GXP tests upfront to detect rifampicin resistance [GXP test is mainly used for the purpose of RR-TB diagnosis]. The 2016 NTP criteria for classifying TB patients as presumptive RR-TB patients are given in Table 1. Patients with rifampicin resistance (with the GXP test), are enrolled for second line drug treatment (also known as MDR-TB treatment) as per the national PMDT guidelines^6^.

**Table 1:** GXP testing Criteria among first line TB patients.

(1) All retreatment TB patients
(2) All new smear positive TB patients
(3) All non-convertor TB patients (i.e., TB patients whose sputum smear is still positive at the end of intensive phase of TB treatment),
(4) HIV seropositive TB patients,
(5) TB patients with past history of close contact with a known MDR-TB patient and
(6) TB patients with diabetes mellitus.

TB-Tuberculosis; HIV- Human Immunodeficiency Virus; MDR-TB-Multidrug Resistant TB.

There are two more sub-groups of patients who are not included in township TB register and are also eligible for GXP testing. They are: a) HIV seropositive patients with TB symptoms; and b) certain cases to be considered individually by MDR-TB committee (e.g., persons with TB symptoms with past history of TB treatment)^6^. However, no line list of these “presumptive RR-TB patients” are maintained anywhere and therefore, our study does not include these patients.

**Figure 1:**
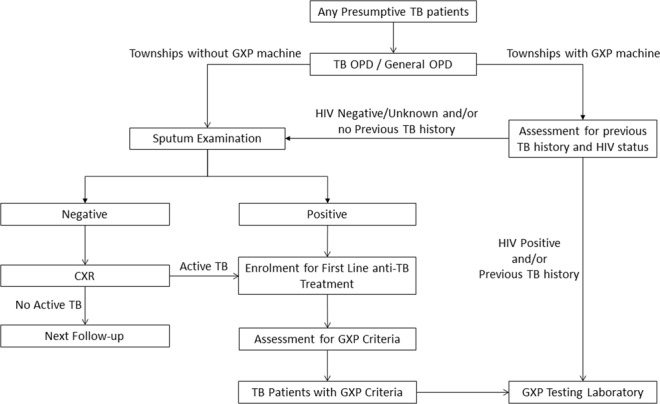
Flow of patients for Xpert MTB/Rif testing in Bago region, Myanmar 2016–2017. OPD—outpatient department; TB—Tuberculosis; GXP—Xpert-MTB/Rif; HIV—Human Immunodeficiency Virus; CXR—Chest X ray.

In Bago Region, there are 28 township health facilities that diagnose and treat TB patients. Each of these 28 townships has a township TB coordinator. It is the primary responsibility of the township TB coordinators and the laboratory technicians to identify patients with presumptive RR-TB and ensure that they undergo GXP testing. GXP testing facilities are available in 4 townships (Bago, Pyay, Taungoo and Taryarwadi) and the sputum samples from non-GXP townships (n = 24) are transported to these four GXP sites for GXP testing. The process followed for GXP testing in Bago region is shown in Fig. 1. In 2016, in townships without GXP testing facilities, the sputum samples were collected and transported to the designated GXP facilities twice a month by township TB coordinators and in 2017, it was transported more frequently on a weekly basis.

### Study population

For the quantitative part of the study, all TB patients enrolled for first-line anti-TB treatment under the NTP between October 2016 and March 2017 in the 28 townships of the Bago Region were included. For the qualitative part of the study, the township TB coordinators (employed and in-place in January 2018) in all 28 townships and TB laboratory technicians from the 4 GXP sites were included.

### Data collection and data validation

For the quantitative part of the study, for each TB patient enrolled in the 28 township TB registers, we applied the criteria given in Table 1 to identify presumptive MDR-TB patients (based on the information recorded in the TB registers). For each patient, their status of GXP test eligibility was assessed at two time points: First, at enrollment and second, at the end of intensive phase of treatment (to identify non-converters). All patients who fulfilled one or more criteria were line listed and this list provided the number of presumptive MDR-TB patients eligible for GXP testing. In order to assess what proportion were tested we looked at the corresponding columns in the TB registers, GXP registers of the four GXP sites and the township TB coordinator’s list (if it was maintained). If there was no information on GXP testing from these three sources, then we classified such patients as “GXP not tested”. For each patient tested by GXP, we assessed whether they were tested before or after initiation of TB treatment.

The data collected from the TB registers included date of enrolment, Township TB number, name of patient, age, gender, name of township, type of TB patients (new/relapse/failure/ loss to follow up/other/transfer in), HIV status (negative/positive/unknown), initial smear result (negative/positive), 2/3 month follow up smear result (negative/positive), GXP number and result.

Details of all eligible presumptive RR-TB patients from paper-based records from 28 townships were entered in a structured data collection format created in EpiData (version 3.1, The EpiData Association, Odense, Denmark). Thereafter, the electronic database was imported to STATA (version 14.2, StataCorp, College Station, Texas, USA) and analyzed.

For the qualitative part of the study, we conducted key informant interviews (KII) with 28 Township TB coordinators and 4 laboratory technicians of the GXP testing sites [total n = 32] without any exclusion. The interview guide (for the qualitative study) was developed based on the preliminary findings for the quantitative study which showed considerable gaps in testing. All interviews were conducted face-to-face and audio recorded. The KII were conducted by one of the two co-authors (KWYK and KTS) with technical guidance of another co-author (Saw Saw). All three co-authors are experienced and trained in TB and qualitative research methods. The interviewers (KWYK and KTS) were not part of NTP but working as Operational Research Fellows for The Union and Department of Medical Research in Myanmar. The KII interview guides are given in Appendix 1.

### Data analysis

#### Quantitative part

Data are summarized in numbers (and proportions) for categorical variables and medians (and interquartile ranges) for continuous variables.

Multivariable log binomial models (multivariable Poisson regression models with robust standard error estimates if log binomial models failed to attain convergence) were used to assess the association between measured demographic and clinical characteristics with lack of GXP testing in those eligible. The association has been described in relative risks and adjusted relative risks with its 95% CI. We did not exclude patients with missing values. Instead, we assigned a value for missing data in each variable and estimated the relative risk and adjusted relative risk for this missing value. For statistical significance we have used a p-value < 0.05.

### Quantitative part

For the data collected through KIIs, we removed the identifying details of participants and their audio files were anonymized. All KII interviews were conducted in Burmese language and transcribed verbatim in Burmese. We did a content analysis. Two coders (KWYK and KTS) independently reviewed all transcripts and developed codes manually. After the codes were developed, emerging themes were identified in consultation with two other co-authors (TO, Saw Saw)—who also had access to all transcripts. The themes are presented as a list of reasons for attrition and a list of possible solutions to address this problem (along with the supporting ‘quotes’). The qualitative data analysis was initially conducted in Burmese and the final results were translated into English. Forward and backward translations of the results were done to ensure accuracy of the translation. The qualitative data analysis was done after the end of all the interviews and we did not check for saturation in-between. We have integrated the results of quantitative and qualitative part of the study through narration^13^.

### Adherence to guidelines

We have adhered to the Strengthening the Reporting of Observational Studies in Epidemiology (STROBE) guidelines and ‘Consolidated Criteria for Reporting Qualitative Research (COREQ) in conducting and reporting the study^14,15^.

### Ethical approval

We obtained permission to conduct the study from the Myanmar NTP. We obtained ethics approval from the Department of Medical Research, Myanmar and Ethics Advisory Group of International Union Against Tuberculosis and Lung Disease (The Union), Paris. Informed consent was obtained from the study participants for the qualitative part of our study. We adhered to the approved methods while conducting the study.

## Results

### Quantitative part

#### Eligible for GXP testing and GXP testing status

A total of 5,658 TB patients were enrolled for TB treatment between October 2016 and March 2017 in Bago Region. Of the six eligibility criteria (shown in Table 1), none of the patients had history of contact with an MDR-TB patient or diabetes mellitus status recorded. Therefore, we were able to assess for GXP eligibility based on four criteria. With this, we found that 2,331 (41%) patients were eligible for GXP testing. The number of patients who fulfilled various eligibility criteria are shown in Table 2.

**Table 2:** The number of patients who fulfilled various eligibility criteria in Bago region, Myanmar, October 2016 to March 2017.

Eligibility criteria	Number
All retreatment TB patients	629
All smear positive TB patients	1741
All non-convertor TB patients	236
HIV seropositive TB patients	241
TB patients with past history of close contact with a known MDR-TB patient	—
TB patients with diabetes mellitus	—

TB—Tuberculosis; GXP—Xpert-MTB/Rif ® tests; HIV—Human Immunodeficiency Virus.

Of those 2,331 patients eligible for GXP testing, 1,066 (46%) had not undergone GXP tests. The demographic and clinical characteristics of all enrolled patients, GXP eligible patients and those who had not undergone GXP tests are shown in Table 3. Adjusted analysis indicated the following: Patients from townships without GXP testing facilities were 6.4 times more likely to not undergo GXP tests when compared to patients from townships with GXP testing facilities. Patients who were new TB cases were 3.8 times more likely to not undergo GXP tests when compared to retreatment TB patients. Patients whose HIV status was negative or unknown were 1.7 times and 2.2 times more likely to not undergo GXP tests when compared to HIV positive patients. Extra-pulmonary TB patients were 2 times more likely to not undergo GXP test when compared to pulmonary TB patients. Additionally, patients aged 45–64 years (when compared to those aged 15 to 44 years) were more likely to undergo GXP test.

**Table 3:** Characteristic of all TB patients enrolled, characteristics of TB patients who were eligible for GXP test and the characteristics of those who did not undergo GXP testing in Bago Region, Myanmar, October 2016–March 2017.

Variable	TB patients enrolled	GXP test eligible patients	GXP not tested	Relative risk	Adjusted relative risk	P value
	N (col %)	N (row %)	N (row %)	(95% CI)	(95% CI)	
**Total**	5,658	2331 (41%)	1066 (46%)			
**Age (in years)**						
<15	1287 (23%)	28 (2%)	19 (68%)	1.36 (1.04–1.76)	1.37 (0.95–1.97)	0.082
15–44	2070 (37%)	1112 (54%)	556 (50%)	Reference	Reference	
45–64	1609 (28%)	874 (54%)	362 (41%)	0.83 (0.75–0.91)	**0**.**88** (**0**.**81–0**.**95**)	**0**.**002**
≥65	692 (12%)	317 (46%)	129 (41%)	0.81 (0.70–0.94)	0.95 (0.84–1.07)	0.394
**Gender**						
Female	2194 (39%)	752 (34%)	370 (49%)	1.12 (1.02–1.22)	**1**.**07** (**1**.**00–1**.**16**)	**0**.**041**
Male	3463 (61%)	1579 (46%)	696 (44%)	Reference	Reference	
Not Recorded	1 (0%)	0 (0%)	0 (0%)			
**Health Facility**						
TB township with GXP machine	1503 (27%)	644 (43%)	64 (10%)	Reference	Reference	
TB township without GXP	4155 (73%)	1687 (41%)	1002 (59%)	5.98 (4.72–7.57)	**5**.**82** (**4**.**62–7**.**32**)	**<0**.**001**
**Type of TB**						
New	5020 (89%)	1701 (34%)	958 (56%)	3.28 (2.75–3.91)	**3**.**69** (**2**.**84–4**.**79**)	**<0**.**001**
Retreatment case	629 (11%)	629 (100%)	108 (17%)	Reference	Reference	
Not recoded	9 (0%)	1 (11%)	0 (0%)	—	—	
**Smear Status and Site of TB**						
Smear Positive Pulmonary TB	1741 (31%)	1741 (100%)	917 (53%)	2.44 (2.06–2.89)	0.79 (0.60–1.04)	0.105
Smear Negative Pulmonary TB	2740 (48%)	524 (19%)	113 (22%)	Reference	Reference	
Extra-pulmonary TB	345 (6%)	27 (8%)	14 (52%)	2.40 (1.61–3.58)	**2**.**07** (**1**.**28–3**.**33**)	**0**.**004**
Not recorded	832 (15%)	39 (5%)	22 (56%)	2.6 (1.9–3.6)	**1**.**56** (**1**.**06–2**.**30**)	**0**.**026**
**Treatment Regimen**						
Initial Regimen	3753 (66%)	1676 (45%)	940 (56%)	3.23 (2.74–3.90)	NA*	
Retreatment Regimen	629 (11%)	629 (100%)	108 (17%)	Reference		
Childhood Regimen	1270 (22%)	25 (2%)	18 (72%)	4.19 (3.11–5.65)		
Not recorded	6 (0%)	1 (17%)	0 (0%)	NE		
**HIV Status**						
Positive	241 (4%)	241 (100%)	85 (35%)	Reference	Reference	
Negative	5019 (89%)	1998 (40%)	935 (47%)	1.33 (1.11–1.58)	**1**.**73** (**1**.**32–2**.**27**)	**<0**.**001**
Unknown	167 (3%)	32 (19%)	17 (53%)	1.51 (1.04–2.18)	**2**.**32** (**1**.**72–3**.**13**)	**<0**.**001**
Not recorded	231 (4%)	60 (26%)	29 (48%)	1.4 (1.0–1.9)	**1**.**73** (**1**.**19–2**.**50**)	**0**.**004**
**GXP Testing Site**						
Pyay	1476	646 (44%)	284 (44%)	0.98 (0.87–1.11)	0.98 (0.88–1.09)	0.668
Tharyarwaddy	558	235 (42%)	97 (41%)	0.92 (0.77–1.10)	0.87 (0.75–1.02)	0.081
Bago	2032	872 (43%)	426 (49%)	1.09 (0.97–1.22)	**1**.**22** (**1**.**12–1**.**34**)	**<0**.**001**
Taungoo	1592	578 (36%)	259 (45%)	Reference	Reference	

*NA: Adjusted RR not estimated for these variables because of multicollinearity or not included in the model.

TB—Tuberculosis; GXP—Xpert-MTB/Rif test; HIV—Human Immunodeficiency Virus; NE = not estimated by the mode.

Of those patients who had undergone GXP test, 26 patients (2% of GXP tested patients) were diagnosed as RR-TB and 23 of them (88% of detected RR-TB) were initiated on MDR-TB treatment. The remaining 3 patients died before initiating MDR-TB treatment (data not shown in the table).

#### Duration between the treatment initiation and GXP testing

In those who had undergone GXP tests (n = 1,265 patients), we assessed whether they had received GXP testing before or after the initiation of TB treatment [Ideally, patients should have GXP testing before initiating TB treatment with first line anti-TB drugs]. Because of the deficiencies in recording dates, for 417 (33%) patients, we could not ascertain whether they had undergone GXP test before or after TB treatment initiation. In the remaining 848 patients, 381 (44%) had undergone GXP tests at least a day after initiating TB treatment (median delay 6 days (IQR: 1–10 days). The delays of these 381 patients disaggregated by demographic and clinical characteristics are shown in Table 4. Patients from townships without GXP machines had longer delays when compared to patients from townships with GXP machines.

**Table 4:** Delays in Xpert MTB/Rif® testing in patients who underwent this test after TB treatment initiation in Bago Region, Myanmar, October 2016–March 2017.

Characteristic	Total (%)	Median days for GXP testing after TB treatment initiation (IQR)
**Total**	381	6 (1–10)
**Age**		
<15 yrs	1 (0.3)	1 (1–1)
15–44 yrs	167 (44)	6 (3–14)
45–64 yrs	148 (39)	6 (3–13)
>/=65 yrs	65 (17)	7 (3–14)
**Sex**		
Female	122 (32%)	6 (3–12)
Male	259 (68%)	7 (2–13)
Health Facility*		
Township with GXP machine	118 (31%)	**3** (**1–7**)*
Township without GXP Machine	263 (69%)	**8** (**4–15**)*
**TB site**		
Pulmonary TB	372 (98%)	6 (3–13)
Extra-pulmonary TB	3 (0.8%)	18 (12–27)
Not recorded	6 (2%)	
**Sputum smear status** (**pulmonary TB cases**)	**372**	
Smear positive	246 (66%)	6 (2–12)
Smear negative	125 (34%)	7 (3–15)
Not recorded	1 (0%)	
**Time of GXP Testing**	**848**	
Before Treatment	390 (46%)	—
On Treatment Date	77 (9%)	—
After Treatment	381 (45%)	—

*Wilcoxon rank sum test p-value < 0.001; TB—Tuberculosis; GXP—Xpert MTB/Rif test; HIV—Human Immunodeficiency Virus.

### Qualitative part

#### Health care provider perspectives for not testing eligible patients with GXP tests

All participants (n = 32) provided consent to participate in KII. The analysis of KII data yielded 6 thematic reasons for not identifying and testing eligible patients for GXP tests and 5 thematic solutions for addressing this issue. The reasons and solutions (along with supporting quotes) are given in Table 5. The most commonly quoted reasons pertained to the themes: unaware of the current guidelines regarding the eligibility criteria for GXP tests, challenges in sputum collection and transportation from patients to the GXP testing sites, human resource constraints (non- availability of additional human resources to cope with the workload) followed by challenges in educating the importance of GXP tests to the patients so that they also make efforts to undergo these tests.

**Table 5:** Health care provider perspectives on reasons for not testing eligible TB patients with Xpert MTB/Rif tests and suggested solutions for improving the situation in Bago Region, Myanmar, January 2018.

Reason for not testing GXP	Solutions suggested
**Reason 1: Unaware of the guidelines** [**GXP testing criteria**] ***Quote****: ‘We did not receive new guideline for GXP criteria* (*2016*). *We follow the existing criteria* (*2015*) *for GXP testing*. *We tested relapse and HIV positive TB patients only’*. (*56-year-old TBC with 7-year service at township with 70% gap for GXP testing*) ***Quote****: ‘The new guideline issued in January*, *2017 arrived at our township TB OPD only at the end of March in 2017’*. (*32-year-old TBC with 2-year service at township with 72% gap for GXP testing*) ***Quote****: ‘When I start working as TBC*, *I did not know the criteria*. *That’s why patients* (*eligible*) *were missed to identify and test*. *[Sometimes] BHS did not know very well about GXP testing criteria*, *even me* (*TBC*) *confused about it’*. (*37-year-old TBC with 1-year service at township with 89% gap for testing GXP*)	**Solution 1: Dissemination of the guidelines** ***Quote****: ‘It is good if they* (*regional office*) *can call me via phone or share the guideline in viber group*. *Then when the guideline arrives to township*, *clerk or data assistant of office share those guidelines to respective department without delay’*. (*56-year-old TBC with 7 years service at township with 70% gap for testing GXP*)
**Reason 2: Shortage of human Resource constraints and high work Load** ***Quote****: ‘I told about GXP testing criteria in monthly meeting*, *but as focal person was not specifically assigned in a decentralized microscopic center*, *many patients were not tested for GXP*. *Even for TB patients*, *proper recording of patients’ information*, *care and tracing could not be done’*. (*23 years old TBC with 1-year service at township with 71% gap for testing GXP)* ***Quote:**** “Lab technician is reluctant to collect and transport more sample for GXP according to new criteria because of limited human resource”*. (*52-year-old TBC with 20-years service at township with 77% gap for testing GXP*) ***Quote:**** ‘It is very bad if the focal person for TB is changed frequently*. *If there is a specific focal person in TB and working only for TB*, *there will be great improvement’*. (*23 years old TBC with 1 year service at township with 71% gap for testing GXP*)	**Solution 2: Dedicated Human resource to support this activity** ***Quote****: ‘There should be one person for laboratory technician*. *Lab technician has to send culture sample to Yangon and send the GXP sample to GXP site as well*. *Even if one person cannot be a lab technician*, *a person should assist him for recording and accepting* *sputum at laboratory’*. (*52-year-old TBC with 20 years service at township with 77% gap for testing GXP*)
**Reason 3: Non-availability of GXP machines in their townships** ***Quote:**** ‘The machine has to be in our township*. *Anyway*, *we can test GXP immediately if there is positive’*. (*33-year-old TBC with 5 years’ service at township with 51% gap for testing GXP*) ***Quote:**** ‘Presence of GXP machine*) *only in Bago is not fine*. *At least*, *GXP machine are provided to townships with high case load should be considered’*. (*Bago*) *5%* (*36 years old TBC with 5 years’ service at township with 5% gap for GXP testing*)	**No solutions proposed**
**Reason 4: Challenges in sputum collection and transportation**. ***Quote****: ‘Some patients sent sputum 2–3 times as per their* (*lab*) *request*. *But as sputum was not sufficient or not in good quality*, *when we asked them to send sputum again*, *they stopped’*. (*33-year-old TBC with 5-years of service at township with 51% gap for testing GXP*) ***Quote:**** ‘The main reason for not testing was patients unable to produce sputum*. *Some patients like smear negative retreated TB patients and patients who had been taking anti-TB treatment for about 1 week*, *cannot produce sufficient amount of sputum*. *Then they did not send the sputum’*. (*34 years old TBC with 1 year service at township with 49% gap for GXP testing*)	**Solution 3: Improve sputum collection and transportation** ***Quote:**** ‘I think it is convenient if patients live in far places*, *they should be asked to collaborate with accessible health staff or NGOs for sending the sputum sample* (*for GXP*)*’*. (*35 years old TBC with 1-year service at township with 69% gap for testing GXP*) ***Quote:**** ‘It is convenient if the volunteer can facilitate in sputum transportation’*. (*35 year old TBC with 4 year service at township with 4% gap for GXP testing*)
**Reason 5: Patients have difficulties in coming to collection sites** ***Quote****: ‘Patients from rural villages were very far from here* (*TB centre*), *they usually take motor-cycle taxi to reach here*, *and it takes 25USD per visit*. *That’s why they didn’t come again to TB centre for sputum examination for GXP’*. (*36 years old TBC with 5-years of service at townships with 5% of gap for testing GXP*)	**Solution 4: Provide incentives or travel allowance to patients** ***Quote:**** ‘If incentive is provided when it* (*sputum sample*) *is sent*, *they* (*patients*) *may come’*. (*42 years old TBC with 1-year service at township with 61% gap for testing GXP*) ***Quote:**** ‘* (*TBC*) *wants to support travel allowance to the person sending the sputum* (*for GXP testing*)*’*. (*39 years old TBC with 1-year service at township with 62% gap for testing GXP*) ***Quote:**** ‘Incentive can be a big issue when it cannot be provided in future*. *It is not sustainable*. *I don’t want to provide* (*incentive*) *to every patient*. *Can government consider providing travel allowance to patients who can submit the letter for proofing poverty?’* (*29-year-old TBC with 1 year service at township with 16% gap for testing GXP*)
**Reason 6: Patients do not understand the importance of GXP test**. ***Quote:**** ‘I told them [patients] that if they tested at private facilities*, *it was very expensive*. *We tested for them free of charge*. *But they never sent their sputum to receive the test’*. (*29 years old TBC with 1-year service at township with 16% gap for testing GXP*)	**Solution 5: Communicate the importance of GXP testing to patients**. ***Quote:**** ‘If we continuously tell patients HE* (*health education*) *and meet them frequently*, *we can test many* (*patients*)*’*. (*47 years old TBC with 2 years service at township with 52% gap for testing GXP*) ***Quote:**** ‘It is mainly our responsibility*. *We failed to communicate them* (*patients*), *they will send if TBC definitely tell them* (*patients*)*’*. (*37 years old TBC with 1 year service at township with 89% gap for testing GXP*)

## Discussion

This is one of the first studies from the Myanmar NTP to assess the magnitude and reasons for pre-diagnosis attrition. The study highlights that 41% of the total TB patients were eligible for GXP testing among TB patients enrolled for first line anti-TB treatment according to 2016 NTP GXP eligible criteria. However, nearly half of them were not tested by GXP. Patient characteristics associated with non-testing were: female sex, patients from TB township without GXP testing facilities, new TB cases, extra-pulmonary TB cases, HIV negative or HIV unknown TB cases. In those who underwent GXP testing, about 44% received the test after initiating TB treatment. The major strength of this study was having a qualitative component to better understand health care provider perspectives on reasons for the programmatic gaps identified in the study.

There were three major limitations of the study: First, since we used routine programme data, there could be errors in recording information on patient care i.e., health care providers may have provided services, but may not have recorded them. Since the programme conducts routine supportive supervisory visits which include on-site verification of records and reports, the errors in recording (if any) are likely to be minimal and random. We strongly feel that programmatic gaps observed in this study, are indicative of the actual gaps in implementation rather than gaps due to errors in recording and reporting information. Second, our analysis and interpretation is restricted to the variables that are routinely collected in the NTPs records. We know that some variables like socio-economic status and distance of the patient’s residence from the health facility can play a major role in eligible patients’ accessing GXP testing services. Since we have not collected information on these variables, we are unable to account for their influence in our analysis. Therefore, the magnitude and direction of association between demographic and clinical variables on GXP testing could be affected by un-measured confounding. Third, in the qualitative part of the study we interviewed only the health care providers and not the patients. Therefore, we are unable to provide the patient perspectives on pre-diagnosis attrition. Moreover, it is possible that health care providers may have given answers that are programmatically desirable. We tried to minimize this by explaining the nature and importance of this research to the study participants and the KIIs were conducted by co-investigators who were not supervisors of the participants.

Despite these limitations, the study’s findings have the following implications on policy and practice.

First, the magnitude of patients eligible for GXP testing was found to be 41% in our study. Since the proportion eligible is highly contextual and is dependent on the local NTP criteria, we are unable to compare this proportion with similar studies from other countries who follow different national eligibility criteria. However, the proportion described in our study could be an underestimate as none of the patients had information on “diabetes mellitus” or “contact with an MDR-TB” recorded in the patient files. This was due to lack of specific ‘fields’ for recording this information in the NTP records. To ensure better implementation of the guidelines and also to facilitate monitoring, we recommend that NTP should revise their recording formats to capture information on all eligibility criteria.

Second, 54% of the patients eligible for GXP testing received the test with 46% pre-diagnosis attrition. Previous studies from India, Bangladesh, Cambodia have reported pre-diagnosis attrition ranging from as low as 17% (in Cambodia) to as high as 60% (in India)^16–19^. The proportion GXP tested in Bago Region (as per our study results was below the 70% target set by the NTP for GXP testing and also falls well below the 90% target set in the End TB Strategy for the detection and treatment of all forms of TB including MDR-TB^20^. This study provides reasons and some solutions to address this gap from the health care providers’ perspective. Of particular concern is the lack of awareness about the current guidelines for GXP testing among health care providers. This could have been avoided by training and better in-house communication and information transmission system among the NTP staff about the current guidelines. The NTP should consider implementing these suggested solutions and monitor the improvements over a period of time. Along with the revisions in patient recording formats, the NTP should revise the reporting format so that cohort information on the number of patients eligible for GXP tests, number tested, and number of RR-TB patients detected and initiated on treatment can be monitored routinely and acted upon.

Third, several patient characteristics were associated with non-testing among GXP eligible patients. From the KIIs it was clear that they were following old NTP criteria, which did not include new smear positive patients, HIV negative or unknown patients for GXP testing. Therefore, these cases were missed. Similarly, patients from townships without GXP testing facilities were facing challenges in sputum collection and transportation. Therefore, the attrition was higher in these townships. Although the reason why attrition was relatively higher in female patients is not clearly known, the relative difference was too small to warrant further investigation. Similarly, only 2% of the TB patients aged (<15 years) were identified as GXP eligible patients when compared to other age groups where nearly 50% of the patients were eligible for GXP-tests. This raises concerns about the adequacy of the present criteria in early detection of RR-TB among younger age groups.

Fourth, by implementing the solutions recommended by the health care providers, gaps in GXP testing for female patients, new TB cases, HIV negative or unknown TB patients could be addressed by training and monitoring health care staff activities. For implementing the solutions to address the gaps in GXP testing for patients from TB townships without GXP testing facilities may require additional financial investment to increase the number of GXP testing facilities and/or improve the sputum collection and transportation systems^21^. However, gaps in GXP testing for extra-pulmonary patients may remain high due to the challenges in collecting appropriate biological specimens for GXP testing (e.g., cerebrospinal fluid, pleural fluid) at peripheral health facilities.

Fifth, in patients who had undergone GXP testing, nearly 44% had this test after being initiated on first line TB treatment. The delay was higher in TB patients from townships without GXP testing facilities. This is primarily due to the practice of fortnightly/weekly sputum collection and transportation from these townships. Although this finding is positive and as per the NTP guidelines that patients are initiated on TB treatment immediately after TB diagnosis (without waiting for GXP result confirmation), it is of concern that patients at high risk for RR-TB initiate treatment without proper diagnosis. In order to address this complex situation, the existing sputum collection and transportation system should be improved while achieving shorter turnaround times.

Lastly, in the qualitative component of the study, issues related to human resource (unavailability of TB focal person in certain townships, frequent turnover of TB focal person) were one of the main reasons for high pre-diagnosis attrition. Allocation of dedicated human resources for TB activities and ensuring their continuous presence is an important solution to narrow this gap.

## Conclusion

In Bago region, 41% of the TB patients enrolled on first line anti-TB treatment were eligible for GXP testing. Pre-diagnosis attrition was high with nearly half of the eligible patients not tested with GXP tests. Training of TB health care providers about the latest GXP testing criteria, improvement in sputum collection and transportation system particularly for townships without GXP testing facilities, allocation of dedicated human resource for TB activities, and enhanced recording, reporting, supervision and monitoring are urgently required to reduce the attrition.

## Supplementary information

Appendix-1

## Data Availability

The data can be obtained from the corresponding author after approval from the National TB Programme and the Department of Medical Research, Ministry of Health and Sports, Government of Myanmar. Anyone interested in the raw data may please contact the corresponding author.
