# Supplementary material for: Magnitude and reasons for pre-diagnosis attrition among presumptive multi-drug resistant tuberculosis patients in Bago Region, Myanmar: A mixed methods study
Source: Sci Rep. 2019 May 10;9:7189. doi: 10.1038/s41598-019-43562-3 (PMC6510739; doi:10.1038/s41598-019-43562-3)
Supplement: Supplementary file 1 — Appendix-1 [file 41598_2019_43562_MOESM1_ESM.docx]

**Appendix-1**

**Title: Magnitude and reasons for pre-diagnosis attrition among presumptive multi-drug resistant tuberculosis patients in Bago Region, Myanmar: A mixed methods study**

**Authors and institutional Affiliations**

Tun Oo^1^, Khine Wut Yee Kyaw^2^, Kyaw Thu Soe^3^, Saw Saw^4^, Srinath Satyanarayana^5^, Si Thu Aung ^6^

1. Assistant Director, National Tuberculosis Programme, Bago Region, Ministry of Health and Sports, Myanmar. E-mail: [dr.tunoo.ntp@gmail.com](mailto:dr.tunoo.ntp@gmail.com)
2. Operational Research Fellow, Department of Operational Research, International Union Against Tuberculosis and Lung Disease (The Union), Myanmar. E-mail: [dr.khinewutyeekyaw2015@gmail.com](mailto:dr.khinewutyeekyaw2015@gmail.com)
3. Research Officer, Department of Medical Research (Pyin Oo Lwin branch), Ministry of Health and Sports, Myanmar. E-mail: [kyawthusoe.dmr@gmail.com](mailto:kyawthusoe.dmr@gmail.com)
4. Director (Planning), Department of Medical Research, Ministry of Health and Sports, Yangon, Myanmar. E-mail: [sawsawsu@gmail.com](mailto:sawsawsu@gmail.com)
5. Deputy Director, Center of Operational Research, International Union Against Tuberculosis and Lung Disease (The Union), Paris, France. E-mail: [ssrinath@theunion.org](mailto:ssrinath@theunion.org)
6. Program Manager, National Tuberculosis Programme, Ministry of Health and Sports, Nay Pyi Taw, Myanmar. Email: [dr.sta.ntp@gmail.com](mailto:dr.sta.ntp@gmail.com)

**Key Informant Interview Guideline for TB coordinators**

District name : _____________________________

Interview Date (dd/mm/yy) : _____________________________

Interviewer’s name (short hand) : _____________________________

TB coordinator identification code : _____________________________

- First introduce yourself, make interviewee comfortable and ensure confidentiality. Introduce study with information sheet and obtain informed consent. If interviewee agrees, proceed to questions.
- Please do not read options to interviewee, except question I)3 and I)4, select the most appropriate option closest to the response.
- Please only select one option for each question, unless specified “select all that apply”
- Please write “NA” for questions F), G), H), I) if TB coordinator is from GXP facilities township
- Please write “NA” for question J) if TB coordinator is from non-GXP sites (24 sites)

**Part A: Structured questionnaire**

1. **Characteristics of TB coordinators**
2. Age of TB coordinator

(_________) years

1. Sex of TB coordinator

Male

Female

1. Serviced years of TB coordinator in recent post

(_________) years

1. **GXP Facility (for all TB coordinators)**
2. Is GXP testing available in your TB center?

Yes

No

1. **Human Resource (for all TB coordinators)**
2. How many number of government staff are working in TB center?

(__________)

1. Does a focal person for TB control activities have other non-TB related activities?

Yes

No

If the answer is “No”, please write “NA” in question B)3

1. If yes, what are the non-TB related activities? (List the activities)

_________________

_________________

_________________

_________________

_________________

1. Is there any staff assigned for collection and sending the sputum samples to GXP facilities?

Yes

No

If the answer is “No”, please write “NA” in question B)5

1. If yes, please provide the position of the responsible staff.

Position: ____________________________

1. **Challenges in convincing the patients to send sputum samples to collection point for GXP testing (for all TB coordinators)**
2. Does the patient or patient’s attendant send the sputum samples to collection point for GXP testing?

Yes

No

1. Is there any assigned person for transportation of patient’s sputum to collection point for GXP testing?

Yes

No

If the answer is “No”, please write “NA” in question C)3

1. If TB coordinator answer Yes in question C)2, who is the responsible person for transportation of patient’s sputum to collection point for GXP testing?

Not applicable

DOT Provider

DOT Supervisor

TB Coordinator

Other, please specify______________

1. Is there any budget for transportation of sputum samples from patient’s home to sputum collection point for GXP testing?

Yes

No

1. **Sputum Quality (for all TB coordinators)**
2. What type of patient mostly produces poor quality sputum or insufficient amount of sputum for GXP testing?

________________________

________________________

________________________

________________________

1. **Schedule for sending sputum samples to GXP facility (for TB coordinators from non GXP sites)**

**Not applicable (NA)**

**Applicable**

1. How often do you collect and send sputum samples to GXP facility?

Not applicable

Weekly

Twice a month

Monthly

Depending on the presence of eligible patient for GXP testing

If the answer is one of the upper three answer in Question E)1., continue to answer Question E)2. and E)3. If the answer in question E)1 is the lowest one, please choose NA in question E)2 and E)3.

1. The minimum number of eligible patient whose sputum samples need to be collected on the schedule date of sending

Not Applicable

At least one eligible patient

At least two eligible patients

At least three eligible patients

At least four eligible patients

1. The maximum time that the patient has to wait for the date of sputum collection point after being enrolled for TB treatment

Not Applicable

One week

Two weeks

Three weeks

Four weeks and more

1. **Sending the sputum sample to GXP facilities (for TB coordinators from non GXP sites)**

**Not applicable (NA)**

**Applicable**

1. Which vehicle do you use for transporting the sputum sample?

Not applicable

Public transportation

Cycle

Bicycle

Other (please specify) _________________

1. How much time do you need to travel to GXP facilities with above your selected option from your township?

_______________Hours

_______________Minutes (if less than 1 hour)

1. **Transportation material (for TB coordinators from non GXP sites)**

**Not applicable (NA)**

**Applicable**

1. Does NTP supply cold box or ice box for samples transportation?

Not applicable

Yes

No

1. How do you transport the sputum samples to GXP facility?

Not Applicable

By Cold box or ice box

Other: ________________

1. **Transportation cost (Financing System) (for TB coordinators from non GXP sites)**

**Not applicable (NA)**

**Applicable**

1. Is the budget allocated for sample transportation?

Not applicable

Yes

No

1. What method is used for covering transportation cost at present?

Not applicable

Advance

Reimbursement

Other, please specify___________________________

1. Which method do you prefer for transportation cost? (Please read the option)

Not applicable

Advance

Reimbursement

1. Which system do you prefer for sending sputum sample to GXP facilities? (Please read the option)

Not applicable

Sending the sample by assigned person from township TB center

Sending the sample by patients to GXP facility and reimbursement to patients when the patient return to township TB center with the GXP result

1. When did you get reimbursement from field finance assistant (FFA)

Not applicable

Within one month

Within two months

Within three months

More than three months

1. **Laboratory workload of GXP facility (for TB coordinators from GXP sites)**

**Not applicable (NA)**

**Applicable**

1. How many rounds of GXP testing does Xpert Machine run per day?

Not applicable

One round

Two rounds

Three rounds

Four rounds

1. Do you need to get appointment with non GXP facilities site for receiving the sputum samples?

Not applicable

Yes

No

**PART B: Open questionnaire**

1. What is your opinion on MDRTB causes, prevention, diagnosis and treatment? **(Participant’s Opinion)**
2. What are the eligible criteria for GXP testing? **(Participant’s knowledge)**
3. What are the barriers that you experienced in sputum collection for GXP testing? What have you done to overcome those barriers? **(Participant’s Opinion)**
4. What are the barriers that you experienced in sending the sputum sample to GXP facilities? What have you done to overcome those barriers? **(Participant’s Opinion)**
5. What will you do if a patient cannot produce sufficient amount of sputum for GXP testing? **(Current practice)**
6. What are the most common reasons for not testing GXP? **(Participant’s Knowledge)** How can we do it better?
